# Assisted life termination and truth telling to terminally ill patients – a cross-sectional study of public opinions in Israel

**DOI:** 10.1186/s13584-020-00419-9

**Published:** 2020-10-26

**Authors:** Moran Bodas, Baruch Velan, Giora Kaplan, Arnona Ziv, Carmit Rubin, Kobi Peleg

**Affiliations:** 1 Israel National Center for Trauma & Emergency Medicine Research, The Gertner Institute for Epidemiology and Health Policy Research, Sheba Medical Center, Tel Hashomer, 5265601 Ramat-Gan, Israel; 2 The Department of Emergency Management & Disaster Medicine, School of Public Health, Sackler Faculty of Medicine, Tel-Aviv University, Tel Aviv-Yafo, Israel

**Keywords:** End-of-life, Doctor-assisted-death, Truth-telling, Autonomy

## Abstract

**Background:**

End-of-life decisions are highly complex socio-normative and ethical phenomena. The goal of this study was to provide an assessment of public opinions in Israel concerning aspects of end-of-life decisions.

**Methods:**

An online cross sectional study was performed in February 2020. The primary tool including items pertaining to death assistance and truth telling to patients. A sample of 515 participants representative of the adult Israeli population was obtained.

**Results:**

The majority of participants (71%) supports telling the entire truth to patients even in harsh conditions. Support for truth telling decreases with affiliation to religion, with as little as 40% support among ultra-orthodox. People with vocational education are the least supportive of truth telling. Concerning doctor assisted death, almost half (49%) of the sample were supportive. Opposition is positively associated with religiosity, with 90% of ultra-orthodox and 58% of religious participants opposing doctor-assisted death, compared to only 18% among seculars. Non-Jews were 3.35 times (95%CI: 1.90, 5.91) more likely to oppose doctor assisted death than Jews (*p* < .0001). An Interrelationship analysis crossing between attitudes revealed that the largest group (39%) was comprised of participants who support both (“autonomists”).

**Conclusions:**

Israelis are overwhelmingly supportive of truth telling to patients. In contrast, Israeli public opinions on doctor assisted death are divided. For both attitudes, religiousness plays a crucial role as a catalyst for conservatism and opposition to change. Almost a half of the public is also supportive of an autonomist approach that would allow patients to decide on ending their own lives.

## Introduction

End-of-life decisions and processes are becoming more complex in recent years due to shifts in socio-normative and ethical perceptions. Terminally ill patients and the caregivers are faced with a multitude of related issues, including the desire and ability of the patient and his or her family members to accept the truth about their medical condition, as well as the physicians’ hardships in telling it. This is often related to the patient’s- readiness to accept fate and to be willing to shift toward palliative care. Moreover, the level of involvement of the physicians in the final stages of life, the legitimacy of active death assistance procedures or cessation of life support, etc. are becoming part of the medical debate in recent years [1–4].

Lately, the awareness of the Israeli public to the psychosocial aspects of dealing with incurable diseases and end-of-life process through palliative care is increasing [4]. A national plan for palliative care was established in 2015 at the request of the Ministry of Health. According to this plan, palliative care should be perceived as part of an overarching care system designed to improve terminally ill patients’ coping capacities and quality of life. Nevertheless, and despite these improvements, the extent of palliative services offered in Israel is “poor, far from meeting the needs of the population, and does not compare to the services provided in advanced countries in the world.” [5].

The legal statute in Israel concerning the medical treatment of terminally ill patients is rooted in the Patient’s Right Act (1996) and the Dying Patient Act (2005). The latter, in particular, allows a person defined as a “dying patient” to request certain treatment or deny others. The medical team is obliged to honor the patient’s will, unless in cases involving the prevention of treatment designed to relief pain and suffering or procedures aimed at assisting death. This setting bring forward numerous questions, including whether the legal status in Israel actually reflects public opinions regarding the issue at hand. Additionally, one may ponder whether the abovementioned legal and formal framework in Israel is compatible with the steadily increasing favorable views of end-of-life processes by the public in Israel and abroad. Before these questions can be answered, a review of global status is required.

The study on public opinion on end-of-life decisions and processes can be traced back to the late 1940s [6]. It gained momentum during the 1990’s [7, 8]. For instance, in 1999, a study of 1885 medical personnel, military veterans, family members of patients who recently died from an incurable disease, and their physicians, found some commonalities between these groups. For example, all four groups rated as being important concerns related to pain and symptom management, preparation for death, achieving a sense of completion, decisions about treatment preferences, and being treated as a “whole person.” [9] An earlier study in the United States found inter-sectorial differences in truth telling to terminally ill patients and their family’s decision to avoid artificial life support [10].

Similar socio-cultural gaps in perception of end-of-life processes were found in other countries. For example, a study performed in 2014 among 68,000 participants from 47 European countries, suggested a polarization of west and east Europe concerning the use of euthanasia in terminally ill patients, with western countries generally tending toward approval of such procedures [11]. In general., support of death assistance procedures can be found mostly in more liberal western countries, e.g. New-Zealand, in which 80% expressed their support of euthanasia [12], Belgium [13], Finland [14], or Austria [15]. Nevertheless, some support can be found also in less developed countries, such as India [16] or Iran [17, 18].

Studies have also demonstrated that socio-demographic background may affect the perception of end-of-life decisions. For instance, gender-based differences were reported, with elderly women tending to demonstrate a lesser degree of will to live then elderly men [19]. Another robust finding across many studies is the negative association between affiliation to religion (religiousness) and rejection of euthanasia [11, 14, 20–22]. Scholars agree that opinions concerning end-of-life decisions and processes are influence by a multitude of socio-psycho-cultural factors [11].

The research revolving around end-of-life processes has also assessed physicians and medical teams’ perceptions and attitudes. As early as 1998–1999, Carmel reported gaps in attitudes between patients and medical students and physicians with regards to end-of-life treatment of patients in Israel. According to her findings, physicians were more prone to use life-sustaining treatment at a higher level compared to that requested by the patients [23, 24]. Nevertheless, Carmel’s finding also suggest trend changes, with an increase in patients’ will to avoid artificial extension of their lives, and even an increase in elderly patients’ consent for palliative care and patient-initiated death assistance [1, 25].

Physicians and medical caregivers seem to be more conservative in their approach to end-of-life decisions [26, 27]. However, this seems to be changing with the progression of time. During 2018, about 3000 physicians in Israel were surveyed for their opinions about death assistance and truth telling to patients. Only half (47%) of the participants indicated that the entire truth should be told to a terminally ill patient. Interestingly, over a half (55%) expressed their willingness to assist a patient requesting to end their life in spite of the current legislation which forbids it, About 30% said they would refuse such a request and the remainder of participants were unable to determine [4, 28].

The findings of prior research in the field of end-of-life processes illustrate the complexity of this topic and highlight the importance of further exploration. It is important to understand the public opinions concerning death assistance and truth telling to support the designing of national policies. Therefore, the goal of this study was to provide current assessment of public opinions in Israel with regard to the abovementioned aspects of end-of-life decisions and processes.

## Methods

### Aim, design and study setting

The goal of this study was to provide an assessment of public opinions in Israel concerning aspects of end-of-life decisions. A cross sectional study was performed in February 2020 with the assistance of an online polling company called iPanel. Since 2006, the iPanel provides an online platform for a wide variety of information collection services, including polls and public opinion surveys. With a pool of more than 150,000 panelists, the iPanel allows rapid access to representative samples of the adult population in Israel through online surveys. iPanel adheres to the stringent standards of the world association for market, social, and opinion researchers (ESOMAR).

### Population and sample

The population for this study included all adult (≥18 years) citizens of the State of Israel. The minimum sample size for this population of ~ 9,000,000 (with 95% confidence level and 5% margin of error) is 385 [29]. The final sample of 515 participants was representative of the target population. See Table 1 for the breakdown of the socio-demographic distribution of the final sample.

**Table 1:** Attitude towards truth telling to patients about their medical condition among different socio-demographic subgroups in the survey sample (*N* = 515)

Participants characteristics	Support Telling Entire Truth (STET) N (%)	Oppose Telling Entire Truth (OTET) N (%)	No position N (%)	***P*** value Chi-Square
**All respondents** (515)	361 **(70.9)**	97 **(19.1)**	51 **(10.0)**	
**Gender**				0.18
Male (*n* = 251)	184 **(73.3)**	48 **(19.1)**	19 **(7.6)**	
Female (*n* = 258)	177 **(68.6)**	49 **(19.1)**	32 **(12.4)**	
**Age**				0.35
18–34 (*n* = 195)	136 **(69.8)**	41 **(21)**	18 **(9.2)**	
35–44 (*n* = 104)	80 **(76.9)**	19 **(18.3)**	5 **(4.8)**	
45–54 (*n* = 74)	55 **(74.3)**	9 **(12.2)**	10 **(13.5)**	
55–64 (*n* = 61)	41 **(67.2)**	12 **(19.7)**	8 **(13.1)**	
65 ^a^ (*n* = 75)	49 **(65.3)**	16 **(21.3)**	10 **(13.3)**	
**Education** (years)				<.0001
< K12 (*n* = 55)	41 **(74.6)**	12 **(21.8)**	2 **(3.6)**	
K12 (*n* = 139)	103 **(74.1)**	29 **(20.9)**	7 **(5.0)**	
> K 12 nonacademic (vocational) (*n* = 112)	58 **(51.8)**	32 **(28.6)**	22 **(19.6)**	
> K12 academic (*n* = 201)	158 **(78.6)**	24 **(11.9)**	19 **(9.5)**	
**Income**				0.05
Bellow average (*n* = 198)	131 **(66.2)**	47 **(23.7)**	20 **(10.1)**	
Average (*n =* 112)	87 **(77.7)**	21 **(18.7)**	4 **(3.6)**	
Above average (*n =* 139)	106 **(76.3)**	20 **(14.4)**	13 **(9.3)**	
**Religiousness**				<.0001
Secular (*n* = 211)	160 **(75.8)**	32 **(15.2)**	19 **(9.0)**	
Tradition keeper (*n* = 152)	120 **(79.0)**	23 **(15.1)**	9 **(5.9)**	
Religious (*n* = 92)	59 **(64.1)**	24 **(26.1)**	9 **(9.8)**	
Ultra-orthodox (*n* = 52)	21 **(40.4)**	18 **(34.6)**	13 **(25)**	
**Ethnicity**				0.33
Jewish (*n* = 410)	285 **(69.5)**	81 **(19.8)**	44 **(10.7)**	
Different^a^ (*n* = 99)	76 **(76.8)**	16 **(16.2)**	7 **(7.0)**	

^a^Muslim, Christian, and Druze

### Tools and variables

The primary tool used in this study was a questionnaire including two items presenting contemporary ethical dilemmas – one pertaining to death assistance and another pertaining to truth telling to patients about their medical condition. The phrasing of the items highlighted the ethical dilemma aspect of each. The death assistance question was phrased as follows: “In your opinion, should a physician assist a terminally ill patient to end his/her life, if the patient asks for it and their medical condition justifies it (i.e., they have very little time to live and they are suffering), providing, of course, that the law would allow that?”. The possible answers to this question were: (a) “Yes, the physician should assist, because a physician who is responsible for a patient should also help him/her in their end of life”, (b) “No, the physician should not assist, because a physician cannot be part of ending someone’s life”, (c) “I cannot choose between the options”, and (d) “Refuse to answer”.

The truth telling question was phrased as follows: “In your opinion, should a physician tell a patient the entire truth about his/her medical condition, even in harsh and hopeless conditions?”. The possible answers to this question were: (a) “Yes, the physician has to tell a patient the entire truth about the condition, because this is the patient’s right”, (b) “No, the physician does not have to tell a patient the entire truth, if the physician thinks that knowing the truth will harm the patient”, (c) “I cannot choose between the options”, and (d) “Refuse to answer”.

Accordingly, the study had two primary outcomes. The first is the distribution of the responses to the death assistance question and the second is the distribution of the responses to the truth telling question. Socio-demographic variables, including sex, age, ethnicity, religiousness, education, income, etc. were also collected.

### Statistical analysis

Distributions of participants' opinions were summarized according to socio-demographic categories of socio-demographic variables as number and percentage, i.e.: age (18–34, 35–44, 45–54, 55–64 and 65+), sex (male vs female), education (less than12 years, high-school diploma), income (below average, average and above average), religiousness (secular, traditional, religious, ultra-orthodox), ethnicity: (Jewish vs non-Jewish). The Chi-square test was used to compare distribution across categories.

Additionally, Multinomial regression analyses were performed to determine the association between the subjects’ choice of answer to the questions of death assistance and truth telling and the above demographic variables, using the logistic procedure. Positive answer to the questions was set as the reference category for both outcomes. Variables that were found to be insignificant in a level of *p* < 0.05 were removed by a backward elimination method.

A univariate analysis to examine the relation of each explanatory variable with the outcomes, while adjusting for age and sex, was performed. Variables which were significant up to the level of *p* = 0.2, were considered as candidates for the multivariable model. Data analysis was carried out using SAS for unix 9.4.

## Results

### Telling the entire truth to patients

In the overall sample, the majority of participants (71%) support telling the entire truth to patients by their treating physicians even in harsh and hopeless conditions. Only 19% opposed telling the entire truth and the remainder (10%) were undetermined. No statistically significant differences were observed between the preferences of ethnicities, gender, and age groups. Table 1 summarizes the outcomes of the analysis of this item.

Supporting truth telling to patients seems to decline with the increase in religiousness, although the data suggest mixed results. Among Jews, a negative association of religiousness and truth telling support is apparent with ultra-orthodox showing the lowest support for truth telling (~ 40%). Among non-Jews (Muslim and Druze), tradition-keepers seem to be more supportive of truth telling (~ 90%) than secular and religious participants (~ 75 and 56.5%, respectively). There was no difference between religious non-Jews and religious Jews (including ultra-orthodox) in support of truth telling (55.4% vs 56.5%, respectively; *p* = 0.58).

Interestingly, no difference was found between high school educated and university educated individuals. The significant difference between education subgroups is attributed to the group of participants with vocational background (more than 12 years of education but without academic degrees). This group demonstrates a decrease in the support rate for truth telling (~ 52%) compared to all other groups (> 75%).

A multivariate logistic regression analysis of attitude to truth telling, with support being the reference category, was carried out (Table 2a). The results of the logistic regression suggest that religiousness and education are predictors of supporting truth telling, even when adjusted to age and gender. Ultra-orthodox are 3.55 times (95%CI: 1.57, 8.07) more likely to oppose truth telling compared to seculars (*p* = .002). Interestingly, Ultra-orthodox are 4.54 times (95%CI: 1.69, 12.17) more likely to be undetermined about truth telling compared to seculars (*p* = .003). Similarly, individuals with vocational education (more than 12 years without academic degree) are 3.04 times (95% CI: 1.58, 5.85) more likely to oppose truth telling than individuals with academic degrees (*p* = .0008).

**Table 2:** Results of multinomial logistic regression analysis of attitudes to (a) truth telling and (b) doctor assisted death among sample (*N =* 515) with support as a reference group

parameter	Effect	category	Point Estimate	95% Wald		***p*** value	
				Confidence Limits			
(a) **Attitudes toward Truth Telling**							
Religiousness						0.0009	
	Ultra-orthodox vs. Secular	Oppose	3.5550	1.5660	8.0730		0.0024
	Ultra-orthodox vs. Secular	Undetermined	4.5400	1.6930	12.1730		0.0026
	Religious vs. Secular	Oppose	2.1160	1.0960	4.0870		0.0256
	Religious vs. Secular	Undetermined	1.7680	0.7130	4.3810		0.2184
	Traditional vs. Secular	Oppose	0.9000	0.4860	1.6680		0.7386
	Traditional vs. Secular	Undetermined	0.7430	0.3090	1.7870		0.5074
Education						0.0018	
	K12 non-academic vs. K12	Oppose	3.0440	1.5830	5.8530		0.0008
	K12 non-academic vs. K12	Undetermined	2.2730	1.0620	4.8640		0.0344
	Academic vs. K12	Oppose	1.8460	0.9960	3.4190		0.0514
	Academic vs. K12	Undetermined	0.5340	0.2120	1.3450		0.1830
	<K12 vs. K12	Oppose	1.7380	0.7700	3.9220		0.1835
	<K12 vs. K12	Undetermined	0.3420	0.0740	1.5820		0.1698
Age						0.632	
	35–44 vs 18–34	Oppose	1.0370	0.5400	1.9900		0.9133
	35–44 vs 18–34	Undetermined	1.7950	0.6090	5.2920		0.2890
	45–54 vs 18–34	Oppose	0.5880	0.2370	1.4640		0.2540
	45–54 vs 18–34	Undetermined	2.2770	0.6900	7.5150		0.1768
	55–64 vs 18–34	Oppose	1.2390	0.5180	2.9660		0.6305
	55–64 vs 18–34	Undetermined	2.8430	0.8250	9.7950		0.0978
	65^a^ vs 18–34	Oppose	1.2360	0.5410	2.8250		0.6150
	65^a^ vs 18–34	Undetermined	2.4080	0.7150	8.1030		0.1559
Gender						0.2851	
	Female vs. Male	Oppose	1.1410	0.6920	1.8800		0.6054
	Female vs. Male	Undetermined	1.7160	0.8750	3.3670		0.1164
(b) **Attitudes toward doctor assisted death**^a^							
Ethnicity						<.0001	
	Non-Jew vs. Jew	Oppose	3.35	1.90	5.91		<.0001
	Non-Jew vs. Jew	Undetermined	2.78	1.39	5.55		0.0037
Religiousness						<.0001	
	Religious vs. Secular	Oppose	6.6260	3.4790	12.6190		<.0001
	Religious vs. Secular	Undetermined	3.0780	1.3590	6.9720		0.0070
	Traditional vs. Secular	Oppose	1.9310	1.1280	3.3050		0.0164
	Traditional vs. Secular	Undetermined	1.7000	0.8950	3.2300		0.1053
Age						0.2654	
	18 to 34 vs 65^a^	Oppose	3.9570	1.5630	10.0200		0.0037
	18 to 34 vs 65^a^	Undetermined	1.6310	0.6400	4.1570		0.3050
	35 to 44 vs 65^a^	Oppose	3.8430	1.4550	10.1490		0.0066
	35 to 44 vs 65^a^	Undetermined	1.3150	0.4720	3.6620		0.6005
	45 to 54 vs 65^a^	Oppose	2.6750	0.9460	7.5650		0.0636
	45 to 54 vs 65^a^	Undetermined	1.5300	0.5370	4.3560		0.4257
	55 to 64 vs 65^a^	Oppose	3.4040	1.2030	9.6290		0.0210
	55 to 64 vs 65^a^	Undetermined	1.6520	0.5700	4.7930		0.3553
Gender						0.9940	
	Male vs. Female	Oppose	1.0270	0.6320	1.6670		0.9152
	Male vs. Female	Undetermined	1.0030	0.5570	1.8070		0.9909

^a^Ultra-orthodox are excluded from this analysis due to their overwhelming opposition to euthanasia

### Doctor assisted death

Concerning doctor assisted death, almost half (49%) of the sample was supportive, 37% opposed, and 14% were undetermined. Table 3 summarizes the outcomes of the analysis for this item. Among the ultra-orthodox the support rates drop dramatically to less than 2%. If the ultra-orthodox are removed from the analysis, the overall support rate increases to 54%. Similarly, the support rate increases to 55% among participants with academic degrees and 59% among participants earning above the average salary. Supporting doctor assisted death is also strongly associated with older age, with three quarters of 65+ years old expressing their support. Supporters age averaged at 46.25 years (±16.85 SD) compared to those opposing doctor assisted death who averaged at 37.31 years (±14.25 SD).

**Table 3:** Attitude towards doctor assisted death among different socio-demographic subgroups in the survey sample (*N* = 494)

Participants characteristics	Support Doctor Assisted Death (SDAD) N (%)	Oppose Doctor Assisted Death (ODAD) N (%)	No position N (%)	***P*** value Chi-Square
**All respondents** (494)	241 **(48.8)**	183 **(35.0)**	70 **(14.2)**	
**Gender**				0.31
Male (*n* = 243)	111 **(45.7)**	98 **(40.3)**	34 **(14.0)**	
Female (*n =* 251)	130 **(51.8)**	85 **(33.9)**	36 **(14.3)**	
**Age**				<.0001
18–34 (*n* = 186)	68 **(36.6)**	89 **(47.8)**	29 **(15.6)**	
35–44 (*n* = 100)	47 **(47.0)**	41 **(41.0)**	12 **(12.0)**	
45–54 (*n* = 73)	37 **(50.7)**	24 **(32.9)**	12 **(16.4)**	
55–64 (*n* = 60)	33 **(55.0)**	18 **(30.0)**	9 **(15.0)**	
65 + (*n =* 75)	56 **(74.7)**	11 **(14.7)**	8 **(10.6)**	
**Age**				<.0001
18–44 (*n* = 286)	115 **(40.2)**	130 **(45.5)**	41 **(14.3)**	
45–64 (*n* = 133)	70 **(52.6)**	42 **(31.6)**	21 **(15.8)**	
65 + (*n* = 75)	56 **(74.7)**	11 **(14.7)**	8 **(10.6)**	
**Education** (years)				0.02
< K12 (*n* = 53)	17 **(32.1)**	26 **(49.1)**	10 **(18.8)**	
K12 (*n* = 136)	68 **(50.0)**	54 **(39.7)**	14 **(10.3)**	
> K 12 nonacademic (vocational) (*n* = 108)	49 **(45.4)**	46 **(42.6)**	13 **(12.0)**	
> K12 academic (*n* = 196)	107 **(54.6)**	57 **(29.1)**	32 **(16.3)**	
**Income**				<.001
Bellow average (*n* = 191)	72 **(37.7)**	89 **(46.6)**	30 **(15.7)**	
Average (*n* = 110)	62 **(56.4)**	33 **(30.0)**	15 **(13.6)**	
Above average (*n* = 138)	82 **(59.4)**	40 **(29.0)**	16 **(11.6)**	
**Religiousness**				<.0001
Secular (*n* = 206)	143 **(69.4)**	38 **(18.5)**	25 **(12.1)**	
Tradition keeper (*n* = 149)	73 **(49)**	50 **(33.6)**	26 **(17.4)**	
Religious (*n* = 88)	23 **(26.1)**	51 **(58.0)**	14 **(15.9)**	
Ultra-orthodox (*n =* 49)	1 **(2.0)**	44 **(89.8)**	4 **(8.2)**	
**Ethnicity**				<.0001
Jewish (*n* = 400)	214 **(53.5)**	135 **(33.8)**	51 **(12.8)**	
Other^a^ (*n* = 94)	27 **(28.7)**	48 **(51.1)**	19 **(20.2)**	

^a^Muslim, Christian, and Druze

When broken down according to ethnicity, a gap emerges between Jews and non-Jews. While 54% of Jews support doctor assisted death in certain cases, at the same time only about 29% of Non-Jews expressed their support. Nonetheless, when analyzing the data according to religiousness, a similar trend is visible for both Jews and non-Jews, with a decrease in support of assisted death as affiliation to religion increases. In all religiousness groups, Jews demonstrate higher rates of support than non-Jews do (data not shown).

A multivariate logistic regression analysis of attitude to doctor assisted death, with support being the reference category, was carried out (Table 2b). In this analysis, Ultra-orthodox were excluded from the analysis due to their overarching opposition to euthanasia. The results of the logistic regression suggest that non-Jews are 3.35 times (95%CI: 1.90, 5.91) more likely to oppose doctor assisted death than Jews (*p* < .0001). Similarly, religious individuals are 6.63 times (95% CI: 3.48, 12.62) more likely to oppose doctor assisted death than seculars (*p <* .0001). Tradition seekers are 1.93 times (95% CI: 1.13, 3.31) more likely to oppose doctor assisted death than seculars (*p* = 0.02).

### Interrelationships between attitudes to truth telling and doctor assisted death

In an attempt to examine the interrelations between attitudes toward truth telling and doctor assisted death responses were crossed between the two items assessed. This yielded four combinations (as suggested by Velan et al. [4]): (a) Participants who support truth telling and doctor assisted death (defined as “autonomy seekers”), which accounted for 39% of the sample, (b) Participants who support truth telling but oppose doctor assisted death (defined as “deontologists”), which accounted for 23%, (c) Participants who oppose truth telling but support doctor assisted death (“compassionate pragmatics”), which accounted for 7%, and (d) Participants who opposed both (“cure seekers”), which accounted for 11%. The remainder 21% were undefined, as they refrained from expressing opinions on at least on one of the subjects. Of note, the rate of “autonomy seekers” increases to 54% among secular participants, whereas the rate of “deontologists” increase to 29% among religious participants.

**Fig. 1:**
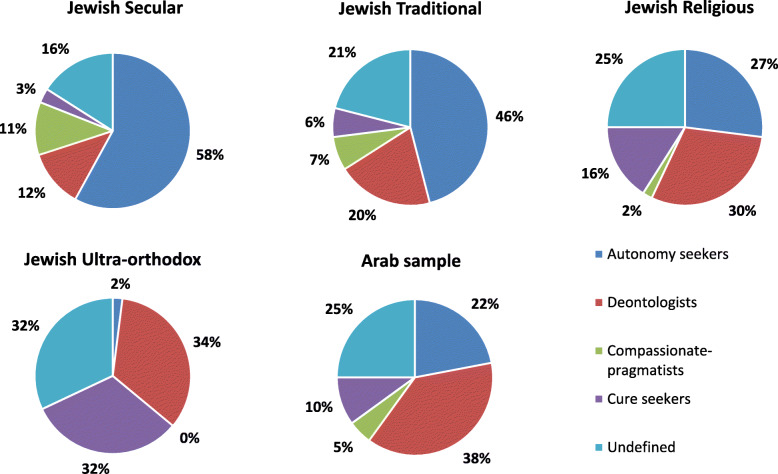
Sociodemographic breakdown of Interrelationships between attitudes to truth telling and doctor assisted death (*N =* 515). *Autonomy seekers*: support both truth telling an doctor assisted death; *Cure seekers*: oppose both; *Compassionate pragmatics*: oppose truth telling but support doctor assisted death; *Deontologists*: support truth telling but oppose doctor assisted death

Figure 1 provides a socio-demographic breakdown of the interrelationships of the studied attitudes. The data shows that while secular Jews are predominantly “autonomy seekers”, the ultra-orthodox are mostly “undefined”, meaning that a significant proportion of the ultraorthodox is undetermined about one or both attitudes assessed. Of the opinionated ultra-orthodox participants, the cure seekers are the predominant group.

Among the non-Jewish (Arab Muslim, Arab Christians, and Druze), the frequency of autonomy seekers is reduced in comparison to the Jewish cohort. Among secular non-Jews, 33% are autonomy seekers, 27% are deontologists, 7% are compassionate-pragmatists and cure seekers (each), and 26% are undefined. Autonomy seekers rate drops with affiliation to religion to 19% among traditional non-Jews and 13% among religious non-Jews. Tradition seeker non-Jews are predominantly deontologists (50%). Religious non-Jews either are deontologists (30%), cure seekers (30%), or undefined (27%).

## Discussion

The results of this study demonstrate a wide public support of truth telling to patients about their medical status, even in harsh conditions. Declared support of truth telling to patient is high across all socio-economic classes, at any age, as well as across ethnic groups (Jews versus non-Jews). The findings here suggest a pan-human inclination to favor truth knowing about a given medical status, even in harsh conditions. This aspect of end-of-life decisions can be considered a consensus among the Israeli public. It is important to note that truth telling is far more supported by the public (~ 70%) than by Israeli physicians (~ 47%) [4], suggesting that there is a wide gap between the public desire to know the truth about their medical condition and the physicians’ willingness (perhaps difficulty) to share this information with terminally ill patients [30–34].

While truth-telling to terminally-ill patients seems to enjoy a consensus among adult Israelis, this cannot be said for the other component of end-of-life decisions assessed in this study – assisted death. This should come as no surprise, as telling or denying the truth from patients may not be equally perceived as taking action to terminate passively or actively a patient’s life. Indeed, the results of this study show a polarization of opinions concerning doctor assist death for terminally ill patients. While almost half of the Israeli public declares support for the notion of active assistance in death for terminally ill patients who wish for this to be done, the opinions concerning doctor assisted death are negatively association to religiousness, as was demonstrated in other studies before [11, 14, 20, 21]. Health-care professionals, families and patients who are religious are less inclined to favor euthanasia and will frequently want medical treatment to be more exhaustive in an effort to preserve life [21, 35, 36]. Indeed, when ultra-orthodox Jews are excluded from the analysis, the data suggests a more favorable stance (~ 58%) toward assisted death practices, which are comparable to those reported by Velan et al. for Israeli physicians [4].

Differences in support of doctor assisted death can be found across demographic groups. For example, ethnicity plays a role in supporting doctor assisted death. According to the data, non-Jews, namely Muslims, Christians and Druze, are more inclined to oppose such procedures (47%) compared to Jews (33%).The data also suggest that the level of education is also positively associated with support of death assisted practices. Lastly, the older the participant and the more they earn, the higher is their support of doctor assisted death. In other words, an older, highly educated, well-earning, secular Jew is most likely to be supportive of doctor assisted death. Having said that, the multivariate regression analysis suggest that only ethnicity and religiousness are predictors of doctor assisted death adjusted to age and gender. While the most forward interpretation suggests that the attitude to doctor assisted death is grounded on theological believes, namely the right of humans to assume the role of the almighty one should keep in mind the strong linkage between religiosity, culture and politics. This is very apparent in Israel where religiosity is associated with conservative views, right wing ideology and even the ancestry, i.e., European Jews (Ashkenazi) and Jews born in Arab countries (Sephardi Jews) [37, 38]. It is possible that the differences observed between different subgroups concerning support of doctor assisted death can be attributed to the multicultural background of the Israeli society, in particular to the sectarianism of the Israeli society between Ashkenazi and Sephardi Jews [39]. This calls for further research to establish the effect of cultural worldviews on attitudes toward end-of-life decisions and process.

The results of the interrelationships between the studied attitudes reveal many important aspects concerning the Israeli public stances toward end-of-life decisions and processes. First, the results suggest that the largest group in the overall population is the autonomy seekers (39%), i.e., those who support both truth telling and doctor assist death. Compared to the data reported by Velan et al. for physicians [4], the results show that the public is more supportive of autonomism than Israeli physicians (28%). Moreover, Lifshitz et al. [40] suggest that there is a gap between family physician knowledge and their performance to empower the persistence of patient autonomy. In contrast, studies exploring similar attitudes among Israeli nurses found a general tendency of nurses to agree with the concept of assisted-death [35, 36]. This finding may suggest that a large portion (more than a third) of the Israeli public is rejecting the paternalism of physicians and seeks more autonomy in end-of-life decisions, as is the case in other countries [41, 42]. .Similar conclusion were reported in other studies. For instance, Hagens et al., who performed in depth interviews with palliative counselees, that having an open non-judgmental attitude, providing trustworthy information and being available are important traits for caregivers of patients who wish to self-determine the timing and manner of their own deaths [43].

Excluding the undefined, the second largest groups are the deontologists, namely people who oppose doctor assist death but support truth telling. Cure seekers who oppose both are only 10% of the population. The results of this analysis suggest that the Israeli public is moving toward a greater support of palliative care, including growing support for end-of-life decisions and process, similar to other western societies [11, 12, 14]. Furthermore, the breakdown of the interrelationships data according to religiousness clearly shows how deontology and cure seeking increase with the increase in affiliation to religion on the expense of autonomism. Interestingly, the undefined group is also increasing with religiousness, suggesting that many religious people are having difficulties addressing the morality of end-of-life processes in terminally ill patients. Perhaps, the data here suggest that there is room for dialogue with religious groups to understand under which circumstances they might be willing to support a greater autonomy to those seeking it, yet are blocked by the current legal framework, which is forbidding euthanasia.

In summary, this preliminary study into Israeli public opinion concerning end-of-life decisions demonstrated that the Israeli public holds favorable views toward truth telling to terminally-ill patients and is polarized with regards to doctor assisted death. Compared with previous reports about Israelis’ opinions [1, 19, 24], the results of the current study suggest a movement toward attitudes that resemble those of western liberal countries. Nonetheless, debates are still raging with regards to whether or not physicians should be involved in assisting patients who so desire to terminate their lives [44]. In any case, this study set the foundations for demonstrating the complexity of this issue in a multicultural society.

### Limitations

This study has several limitations. First, the use of an online survey may cause a bias in assessment of attitudes toward participants with high computer skills. Nevertheless, as discussed in the methodology section, the polling company contracted for the purpose of this study has been demonstrated to be capable of generating statistically representative samples of the Israeli public. Second, as is the case with other cross sectional studies, the results presented in this study are true to the time of collection. Temporal changes in attitudes may have been registered in the specific timing of this study. Third, the multiple choices in the questions utilized for this study are probably not exhaustive, as there may be situations that do not fall within the scope of the case and the possibilities given in the possible answers. Lastly, the study assess participants declared opinion under their current life experience circumstance. These attitudes may differ from actual behaviors when a person is faced with accompanying a loved one on their dying bed.

## Conclusions

Israelis are overwhelmingly supportive of truth telling to patients about their medical status, even in harsh conditions. This is in striking contrast to the proportion of Israeli physicians who support truth telling, which is less than half. This finding calls for further scrutiny of the gaps in attitude between the public and caregivers. In addition, almost a half of the public is also supportive of an autonomist approach that would allow patients who wish for this to be assisted by the doctors in ending their lives. Based on this study and previous ones that assessed Israeli physicians opinions on the subject matter, policy makers may be prompted to consider differential policies for sub groups of the populations to accommodate their wish for greater autonomy in end-of-life decisions. Despite the apparent drift in Israeli public opinion toward a more supportive approach to end-of-life decisions and processes, there remains much to be understood about the effect of cultural worldviews on relevant attitudes. Future research should focus on this aspect, as well as on mapping current practices for end-of-life decisions.

## Data Availability

The datasets used and/or analysed during the current study are available from the corresponding author on reasonable request.
